# Development and Validation of an ADME-Related Gene Signature for Survival, Treatment Outcome and Immune Cell Infiltration in Head and Neck Squamous Cell Carcinoma

**DOI:** 10.3389/fimmu.2022.905635

**Published:** 2022-07-08

**Authors:** Xinran Tang, Rui Li, Dehua Wu, Yikai Wang, Fang Zhao, Ruxue Lv, Xin Wen

**Affiliations:** ^1^ Department of Radiation Oncology, Nanfang Hospital, Southern Medical University, Guangzhou, China; ^2^ The First Affiliated Hospital, Sun Yat-sen University, Guangzhou, China

**Keywords:** head and neck squamous cell carcinoma (HNSCC), ADME, gene signature, survival, immune cell infiltration, treatment outcome

## Abstract

ADME genes are a set of genes which are involved in drug absorption, distribution, metabolism, and excretion (ADME). However, prognostic value and function of ADME genes in head and neck squamous cell carcinoma (HNSCC) remain largely unclear. In this study, we established an ADME-related prognostic model through the least absolute shrinkage and selection operator (LASSO) analysis in the Cancer Genome Atla (TCGA) training cohort and its robustness was validated by TCGA internal validation cohort and a Gene Expression Omnibus (GEO) external cohort. The 14-gene signature stratified patients into high- or low-risk groups. Patients with high-risk scores exhibited significantly poorer overall survival (OS) and disease-free survival (DFS) than those with low-risk scores. Receiver operating characteristic (ROC) curve analysis was used to confirm the signature’s predictive efficacy for OS and DFS. Furthermore, gene ontology (GO) and Kyoto Encyclopaedia of Genes and Genomes (KEGG) pathway analyses showed that immune-related functions and pathways were enriched, such as lymphocyte activation, leukocyte cell-cell adhesion and T-helper cell differentiation. The Cell-type Identification by Estimating Relative Subsets of RNA Transcripts (CIBERSORT) and other analyses revealed that immune cell (especially B cell and T cell) infiltration levels were significantly higher in the low-risk group. Moreover, patients with low-risk scores were significantly associated with immunotherapy and chemotherapy treatment benefit. In conclusion, we constructed a novel ADME-related prognostic and therapeutic biomarker associated with immune cell infiltration of HNSCC patients.

## Introduction

Head and neck squamous cell carcinoma (HNSCC), which originates from the squamous epithelium of the mouth; nasal cavity and paranasal sinuses; oropharynx; larynx and hypopharynx, is one of the most prevalent malignant cancers with more than 800,000 new cases each year (1, 2). Despite multidisciplinary treatment of locally advanced HNSCC by surgery combined with adjuvant chemoradiation or platinum-based concurrent chemoradiation, less than 50% of patients can be cured (3, 4). Tumor-node-metastasis (TNM) staging system helps guide therapeutic decisions and predict clinical outcomes. However, this anatomic staging system does not adequately reflect tumor complexity and heterogeneity in an individual patient. Therefore, identification of effective and sensitive biomarkers for the diagnosis, therapeutic response and prognosis of HNSCC is urgently needed.

Genes involved in drug absorption, distribution, metabolism and excretion are defined as ADME genes (5–7). According to the PharmaADME consortium, ADME genes consist of 32 core genes and 266 extended genes (http://www.pharmaadme.org). These genes are divided into several groups based on their roles in the pharmacokinetic process, such as phase I and II drug-metabolizing enzymes, drug transporters and modifiers (8–10). Growing evidence indicates that ADME gene polymorphism contributes to interindividual variability in carcinogenesis and drug response (11, 12). Consistently, ADME gene expression regulated at the transcriptional, translational and epigenetic levels also shows variability in the population (13–15).

Recent studies have revealed that ADME genes can act as biomarkers to predict therapy response, adverse drug reactions, drug resistance and survival outcomes based on their critical roles in the pharmacokinetic process. Previous research has demonstrated that CYP1B1 and ABCB1 could predict the clinical response to taxane therapy in breast cancer (16). Suthandiram et al. showed that ABCB1 was associated with an increased probability of methotrexate adverse events in patients with haematological malignancies (17). Zhang et al. reported that UGT1A1 expression correlated with 5-fluorouracil resistance in esophageal squamous cell carcinoma (18). Hu et al. identified a set of core ADME genes that could predict overall survival (OS) in many cancer types (19). However, the prognostic prediction values and biological functions of ADME genes in HNSCC have not yet been systematically evaluated.

In this study, we used gene expression data and clinical information from The Cancer Genome Atlas (TCGA) (n = 494) to identify differentially expressed ADME genes in HNSCC. We then defined a 14-gene signature for predicting survival outcomes by the least absolute shrinkage and selection operator (LASSO) analysis in TCGA training cohort (n = 247) and validated it through the internal TCGA validation cohort (n = 247) and an external Gene Expression Omnibus (GEO) cohort (n = 88). Finally, we performed functional enrichment, immune cell infiltration, immunotherapy response, mutation and chemotherapy response analyses between the high-risk group and the low-risk group. Our results indicated that the ADME-related signature was associated with immune cell infiltration and could predict prognosis and therapeutic response in HNSCC.

## Materials and Methods

### HNSCC Dataset Collection

We obtained the gene expression data and clinical annotation of the TCGA-HNSC and TCGA-SKCM datasets from UCSC Xena (https://xena.ucsc.edu/). Patients with no survival information or RNA expression data were removed from further analysis. An independent dataset (GSE102349, n = 113, 88 with survival outcome) from the GEO database (https://www.ncbi.nlm.nih.gov/geo/) was analyzed to validate the prognostic value of the gene signature. Data and clinical information of IMvigor 210 cohort were obtained from the IMvigor210CoreBiologies R package. The probe signal intensity data were log2 transformed and quantile normalized. We transformed the ENSEMBL Gene ID to the Gene Symbol ID. Genes were excluded if they did not express in more than half of the samples. We conducted TCGA and GEO dataset analysis using R software (https://www.r-project.org/).

### Extraction of Differentially Expressed ADME Genes

The 298 ADME genes that are currently defined by the PharmaADME Consortium (http://www.pharmaadme.org) consist of 32 core genes and 266 extended genes (8, 9). Their full names are shown in **Table S1**. Differentially expressed genes between tumor tissues and adjacent normal tissues in the TCGA-HNSC cohort was identified by the ‘limma’ R package. Genes with P value < 0.05 were identified as dysregulated genes. Differentially expressed ADME genes were extracted through VENNY (https://bioinfogp.cnb.csic.es/tools/venny/index.html).

### Identification of a Prognostic ADME-related Gene Signature

We first estimated the prognostic value of the overlapping dysregulated ADME genes through univariate Cox regression analysis. Next a protein–protein interaction (PPI) network for survival-related genes was established through the STRING database (20). We then chose these dysregulated prognostic genes to develop possible risk score through LASSO Cox regression analysis with the ‘glmnet’ R package. Penalty parameter lambda (λ) of the model was chosen according to 10-fold cross validation. The risk score of each patient was calculated according to the normalized expression of the candidate genes (Expi) and their corresponding regression coefficients (Coei). The formula of the risk score was constructed as follows:


$$\text{Risk score}=\sum_{i=1}^{N}\left(Expi\;\times Coei\right)$$

HNSCC patients were stratified into high-risk and low-risk groups according to the median cutoff value. Afterwards Kaplan–Meier and ROC curve analyses were conducted to evaluate the prognostic performance of the novel gene signature with the ‘survminer’, ‘survival’ and ‘survivalROC’ R packages. Finally, univariate and multivariate Cox regression analyses were carried out to estimate prognostic independence of the ADME-related risk score and other clinical parameters in patients with HSNCC.

### Functional Enrichment Analysis

The differentially expressed genes between the high-risk and low-risk groups were defined by the threshold of |fold change| ≥ 1.5 and P < 0.05. These genes were selected to perform gene ontology (GO) analysis and Kyoto Encyclopaedia of Genes and Genomes (KEGG) pathway analysis using the ‘clusterProfiler’ R package. The P-values of GO terms or KEGG pathways were corrected by the Benjamini–Hochberg method. Geneset variation analysis (GSVA) was carried out using the ‘GSVA’ package.

### Estimation of Immune Cell Infiltration

The extent of immune cell infiltration of the tumor microenvironment (TME) in each HNSCC patient was estimated by the Cell-type Identification by Estimating Relative Subsets of RNA Transcripts (CIBERSORT) algorithm (21). The relative abundance of 22 tumor-infiltrating immune cell types (naive B cell, memory B cell, CD8+ T cell, naive CD4+ T cell, resting/activated memory CD4+ T cell, regulatory T cells (Treg), follicular helper T cell, gamma delta T cell, resting/activated dendritic cell, M0/M1/M2 macrophage, eosinophil, neutrophil, resting/activated mast cell, resting/activated NK cell, monocyte and plasma cell) in HNSCC samples was evaluated using the ‘cibersort’ R package. Moreover, the relationship between the risk score and immune cell infiltration was calculated using Spearman correlation analysis, and the results were displayed through the ‘ggstatsplot’ R package.

The stromal score, immune score and ESTIMATE score of each sample were computed through ‘ESTIMATE’ R package (22). The xcell score which reflects the integrative immune activity in the tumor microenvironment was calculated using ‘xcell’ R package (23). Moreover, the other immune response-related scores (CYT score, MHC score, CD8 T effector score) were calculated using the related gene sets according to previous studies (24–26).

### Drug Sensitivity Prediction

Drug sensitivity was estimated through ‘pRRophetic’ R package (27). The ridge regression was performed to calculate the 50% of maximum inhibitory concentration (IC50) based on the Genomics of Drug Sensitivity in Cancer (GDSC) database.

### Statistical Analysis

A Student’s t-test was used to compare the continuous variables and a chi-square test was used to compare the categorical variables. The Kaplan–Meier method was conducted to evaluate OS and DFS, through the log-rank test. Univariate and multivariate Cox proportional hazards regression analysis was carried out to identify independent prognostic factors. A P value <0.05 was considered as significant. The above statistical analyses were performed using R software (Version 3.6.3).

## Results

### Identification of Dysregulated and Prognostic ADME Genes in HNSCC

**Figure 1:**
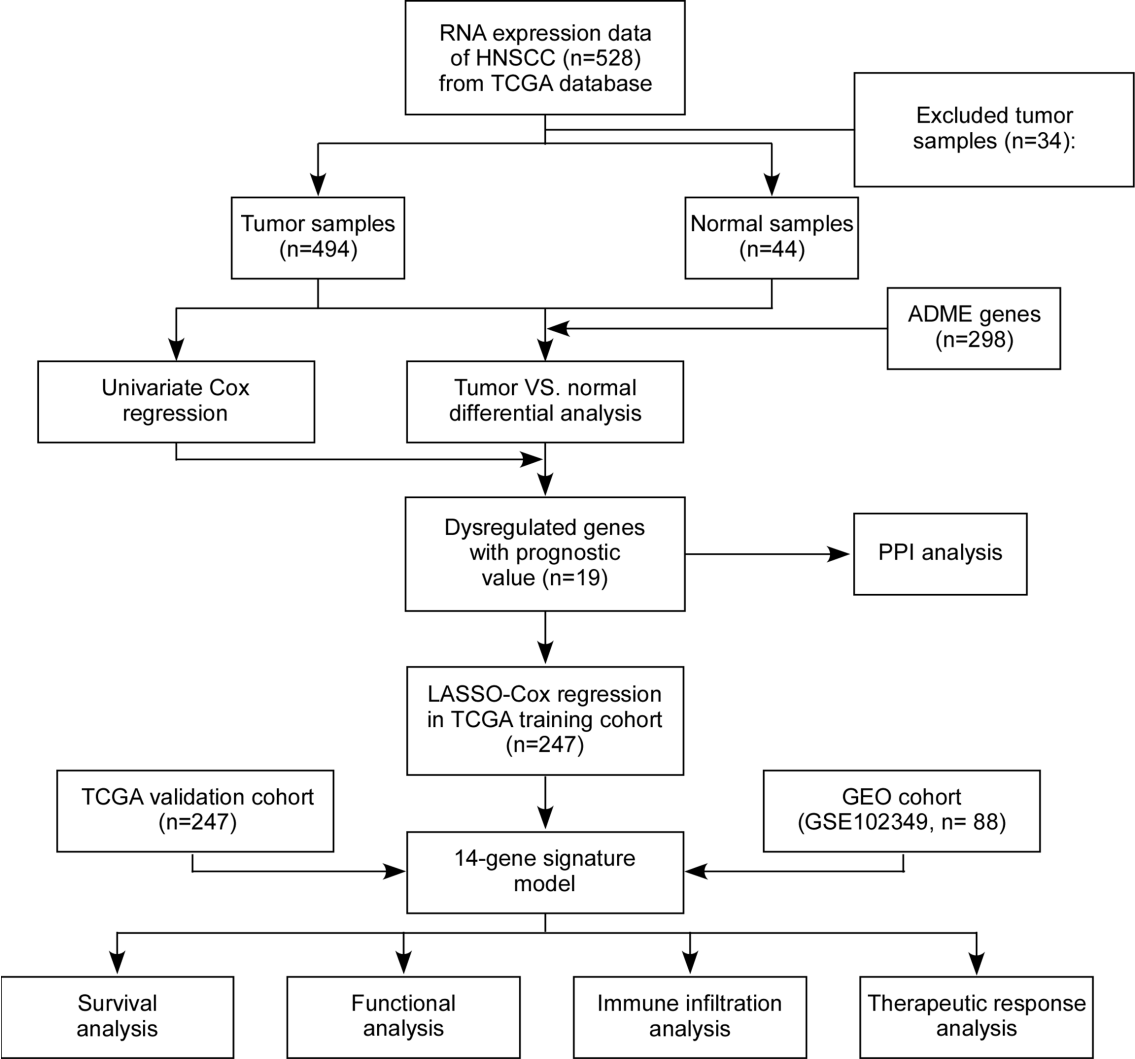
Flow chart of data collection, analysis and validation.

**Figure 2:**
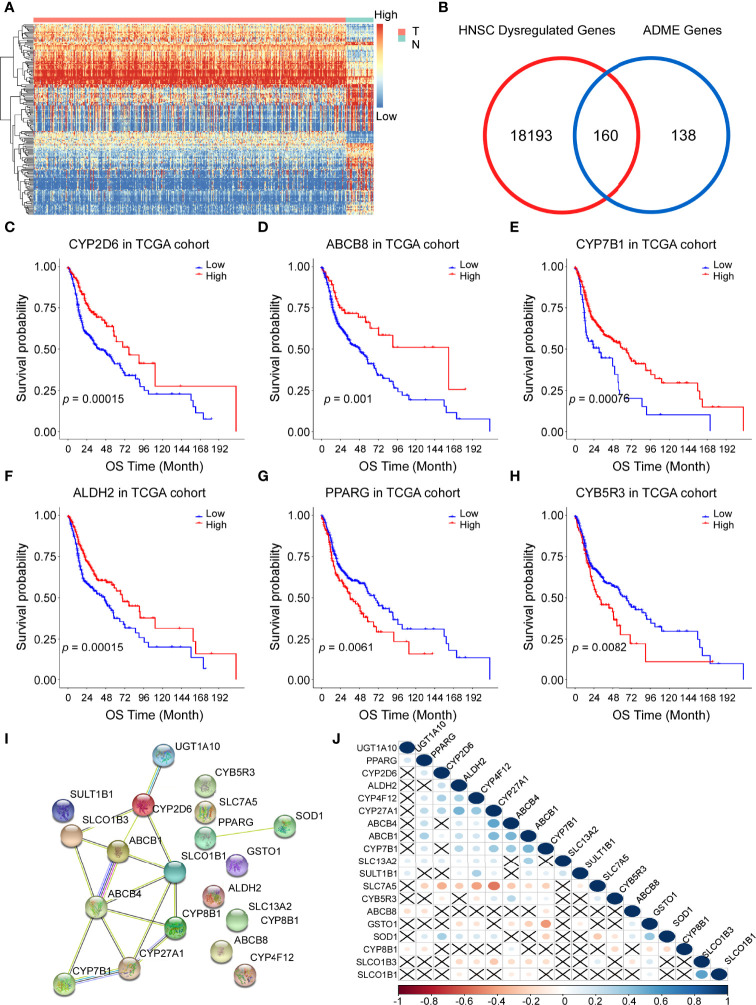
Identification of prognostic genes involved in drug absorption, distribution, metabolism, and excretion (ADME) in the Cancer Genome Atla (TCGA) cohort. **(A)** Heatmap showing the gene expression profiles of tumor (T) and normal (N) tissues. High, high expression; Low, low expression. **(B)** Venn diagram to show differentially expressed ADME genes. **(C–H)** Kaplan–Meier curves of overall survival (OS) for 6 representative prognostic ADME genes in HNSCC, all P <0.01. **(I)** A PPI network indicating the interactions among the 19 prognostic ADME genes. **(J)** Spearman correlation analysis of the 19 ADME genes.

The flow chart of this study is shown in **Figure 1**. We determined the gene expression profiles by comparing HNSCC samples and normal samples in the TCGA cohort and found 18353 dysregulated genes (P < 0.05). The heatmap and volcano plot of differentially expressed mRNAs were presented in **Figure 2A** and **Figure S1**. The Venn diagram showed that more than half of the ADME genes (160/298, 53.6%) were differentially expressed in HNSCC (**Figure 2B**). We performed the univariate COX analysis and identified 19 prognostic ADME genes. The Kaplan–Meier survival curves for the 19 ADME genes in HNSCC are shown in **Figures 2C–H** and **Figure S2** (all P < 0.05). Moreover, we constructed the PPI network of 19 prognostic ADME genes (**Figure 2I**), and the correlation between these genes is presented in **Figure 2J**.

### Construction of a Prognostic ADME Gene Signature in the TCGA Training Cohort

**Figure 3:**
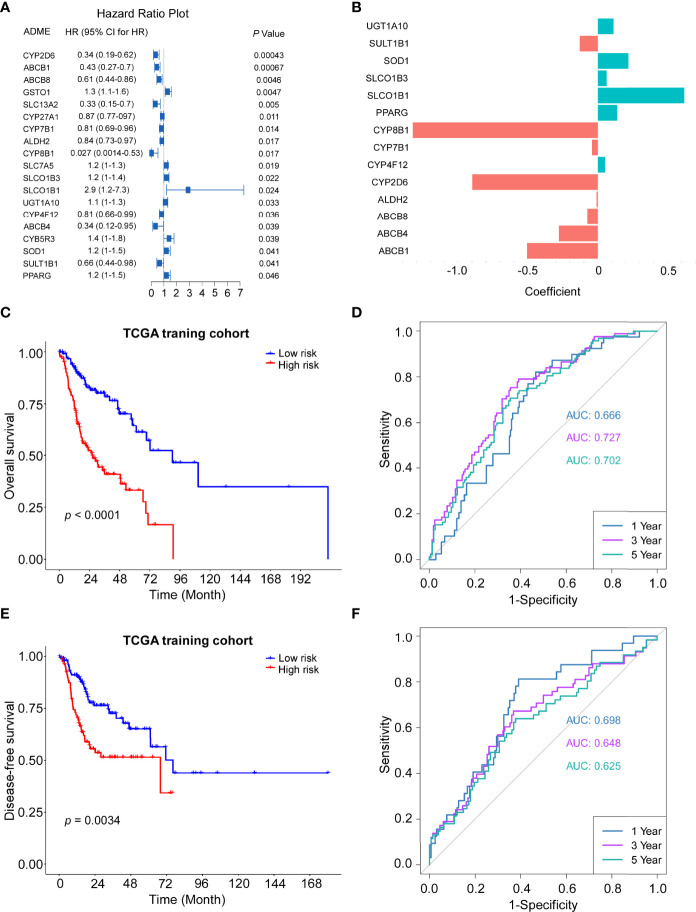
Construction of the 14-gene signature in the TCGA train cohort. **(A)** Forest plot of the univariate Cox regression analysis between expression of 19 ADME genes and OS. **(B)** The coefficients of 14 ADME genes. **(C)** Kaplan–Meier curves for the OS of high-risk and low-risk groups in the training cohort. Log-rank test, P <0.0001. **(D)** Time-dependent ROC curve of risk score in train cohort on OS. **(E)** Kaplan–Meier curves for the disease-free survival (DFS) of high-risk and low-risk groups in the training cohort. Log-rank test, P=0.0034. **(F)** Time-dependent ROC curve of risk score in train cohort on DFS.

To further investigate the prognostic roles of ADME genes, 494 patients of head and neck cancer from the TCGA project were divided into the training cohort (n = 247) and the internal validation cohort (n=247). We conducted LASSO penalized Cox regression analysis using the expression profile of the 19 ADME genes mentioned above (**Figure 3A**) in the TCGA training cohort. We then selected 14 genes (4core ADME genes and 10 extended ADME genes) according to the optimal λ value and the coefficients of candidate genes were derived from the LASSO algorithm (**Figure 3B**). The risk score of each patient was calculated as follows:


$$\begin{array}{l} \text{Risk score}=\;0.112\;\times \text{ UGT}1\text{A }-\;0.129\;\times \text{ SULT}1\text{B}1\;+\;0.223\;\times \text{ SOD}1\;+\;0.062\;\times \text{ SLCO}1\text{B}3\;+\;0.617\;\times \;\\ \text{SLCO}1\text{B}1\;+\;0.138\;\times \text{ PPARG }-\;1.328\;\times \text{ CYP}8\text{B}1\;-\;0.042\;\times \text{ CYP}7\text{B}1\;+\;0.052\;\times \text{ CYP}4\text{F}12\;-\;0.898\;\times \;\\ \text{CYP}2\text{D}6\;-\;0.009\;\times \text{ ALDH}2\;-\;0.076\;\times \text{ ABCB}8\;-\;0.278\;\times \text{ ABCB}4\;-\;0.507\;\times \text{ ABCB}1. \end{array}$$

The HNSCC patients in the TCGA training cohort were stratified into a high-risk group or a low-risk group based on the median cut-off value (0.774). The Kaplan–Meier survival curves indicated that patients in the high-risk group exhibited significantly poorer OS (P < 0.0001) and DFS (P = 0.0034) than those in the low-risk group (**Figures 3C, E**). Time-dependent ROC curves were conducted to evaluate the predictive efficacy of the novel signature for survival. The area the curve (AUC) for OS reached 0.666 at 1 year, 0.727 at 3 years and 0.702 at 5 years (**Figure 3D**). AUC for 1-, 3- and 5-year DFS were 0.698, 0.648 and 0.625, respectively (**Figure 3F**).

### Validation of the 14-Gene Signature in 2 Cohorts

**Figure 4:**
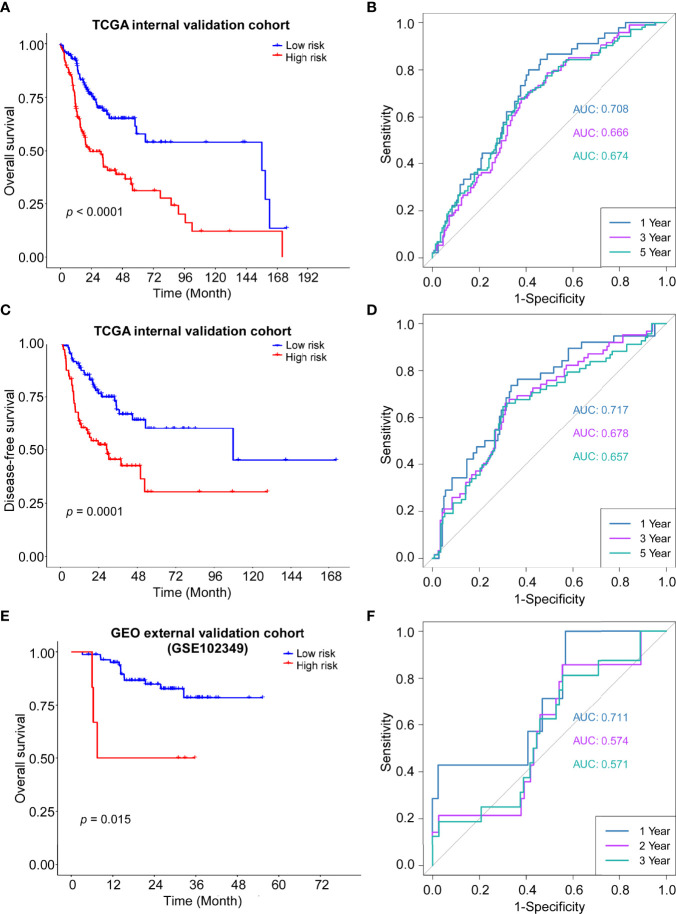
Validation of the ADME gene signature in the internal and external cohort. **(A)** Kaplan–Meier curves for the OS of high-risk and low-risk groups in the internal cohort. Log-rank test, P <0.0001. **(B)** Time-dependent ROC curve of risk score in internal cohort on OS. **(C)** Kaplan–Meier curves for the DFS of high-risk and low-risk groups in the internal cohort. Log-rank test, P=0.0001. **(D)** Time-dependent ROC curve of risk score in internal cohort on DFS. **(E)** Kaplan–Meier curves for the OS of high-risk and low-risk groups in the GSE102349 cohort. Log-rank test, P=0.0015. **(F)** Time-dependent ROC curve of risk score in GSE102349 cohort on OS.

The robustness of the ADME gene signature was tested in the internal validation TCGA cohort (n = 247) and the external validation GEO cohort (GSE102349, n = 88) using the same risk score formula. The validation cohorts were also categorized into high- or low-risk groups by the same cut-off value from the training cohort (0.774). Consistent with the results of the TCGA training cohort, patients in the high-risk group had a shorter overall survival and disease-free survival than those in the low-risk group (**Figures 4A, C, E**). Moreover, ROC curves were applied and the AUC for survival were shown in **Figures 4B, D, F**, respectively.

### Independent Prognostic Value of the 14-Gene Signature

**Figure 5:**
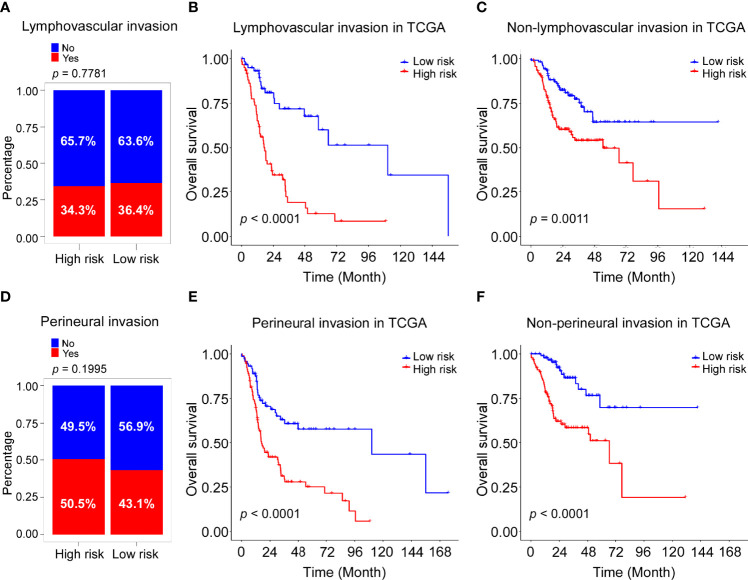
Association of risk score with lymphovascular invasion and perineural invasion in TCGA cohort. **(A)** Rate of patients with lymphovascular invasion (Yes with No) in high and low risk groups in the TCGA-HNSC cohort. **(B)** Kaplan–Meier curves for the OS of high-risk and low-risk groups in the patients with lymphovascular invasion TCGA cohort. Log-rank test, P <0.0001. **(C)** Kaplan–Meier curves for the OS of high-risk and low-risk groups in the patients without lymphovascular invasion TCGA cohort. Log-rank test, P =0.0011. **(D)** Rate of patients with perineural invasion (Yes with No) in high and low risk groups in the TCGA-HNSC cohort. **(E)** Kaplan–Meier curves for the OS of high-risk and low-risk groups in the patients with perineural invasion TCGA cohort. Log-rank test, P <0.0001. **(F)** Kaplan–Meier curves for the OS of high-risk and low-risk groups in the patients without perineural invasion TCGA cohort. Log-rank test, P <0.0001.

The clinical information of the cohort from the TCGA database is shown in **Table 1**. Univariate Cox regression analysis indicated that the risk score, gender, lymphovascular invasion and perineural invasion were all significantly associated with OS (**Table 2**). After adjustment for other confounding factors, the risk score was still correlated with poor overall survival in the multivariate Cox regression analysis (HR = 2.84, 95% CI = 1.92-4.21, P < 0.001, **Table 2**). Chi-square test showed no statistical significance concerning lymphovascular invasion (P = 0.7781) and perineural invasion (P = 0.1995) between different risk groups (**Figures 5A, D**).

**Table 1:** Clinical characteristics of the patients stratified by low and high expression of ADME risk score in TCGA-HNSC cohort.

Characteristics	ADME risk score		P-value
	High	Low	
	(n=240)	(n=254)	
Gender (%)			0.78
Female	66 (27.5)	66 (26.0)	
Male	174 (72.5)	188 (74.0)	
Age (%)			**0.018**
≤ 60	103 (42.9)	137 (53.9)	
> 60	137 (57.1)	117 (46.1)	
Lymphovascular invasion (%)			0.778
No	113 (65.7)	105 (63.6)	
Yes	59 (34.3)	60 (36.4)	
Perineural invasion (%)			0.199
No	90 (49.5)	95 (56.9)	
Yes	92 (50.5)	72 (43.1)	
HPV status (%)			**0.04**
Negative	32 (84.2)	47 (63.5)	
Positive	6 (15.8)	27 (36.5)	
Alcohol (%)			0.095
No	84 (36.2)	72 (28.7)	
Yes	148 (63.8)	179 (71.3)	
Tumor stage (%)			0.681
Stage I-II	52 (21.7)	60 (23.6)	
Stage III-IV	188 (78.3)	194 (76.4)	
Radiation therapy (%)			0.873
No	100 (41.7)	103 (40.6)	
Yes	140 (58.3)	151 (59.4)	
Chemotherapy (%)			0.123
No	167 (69.6)	159 (62.6)	
Yes	73 (30.4)	95 (37.4)	

**Table 2:** Univariate and multivariable Cox regression analysis of prognostic factors in 494 patients with HNSCC.

Variable	Univariate analysis				Multivariate analysis			
	HR	95%CI	*P-*value		HR	95%CI	*P-*value	
Gender (Male vs. Female)	0.73	0.55-0.98	**0.035**		0.89	0.60-1.32		0.57
Age (≤60 vs. >60)	1.02	1.01-0.03	**0.0014**		1.02	1.00-1.04		**0.0052**
Lymphovascular invasion (Yes vs. No)	1.69	1.21-2.38	**0.0024**		1.73	1.19-2.53		**0.0043**
Perineural invasion (Yes vs. No)	2.20	1.55-3.11	**<0.001**		1.95	1.32-2.88		**<0.001**
Hpv status (Positive vs. Negative)	0.52	0.20-1.36	0.18					
Alcohol (Yes vs. No)	0.97	0.73-1.30	0.85					
Radiation therapy (Yes vs. No)	0.82	0.49-1.39	0.47					
Tumor stage (Stage III-IV vs. Stage I-II)	1.17	0.84-1.62	0.35					
Risk score (High vs. Low)	2.05	1.55-2.71	**<0.001**		2.09	1.42-3.07		**<0.001**

The bold p values mean that they are < 0.05.

Stratification analysis was performed to further evaluate whether the ADME-related score was independent of lymphovascular invasion and perineural invasion. Patients in the TCGA dataset were stratified into the lymphovascular invasion group and the non-lymphovascular invasion group. The Kaplan–Meier survival curves showed a significant difference in OS between the high-risk and low-risk groups in the lymphovascular invasion group (P <0.0001, **Figure 5B**) as was in the non-lymphovascular invasion (P = 0.0011; **Figure 5C**). Next, all HNSCC patients were also classified by perineural invasion. Patients with high-risk scores exhibited poorer OS than those with low-risk scores in perineural invasion group (P <0.0001, **Figure 5E**) as well as in non-perineural invasion group (P <0.0001, **Figure 5F**). These results suggest that the novel signature derived from ADME genes is an independent prognostic predictor in HNSCC patients.

### Functional and Immune Infiltration Analyses in Different Risk Groups

**Figure 6:**
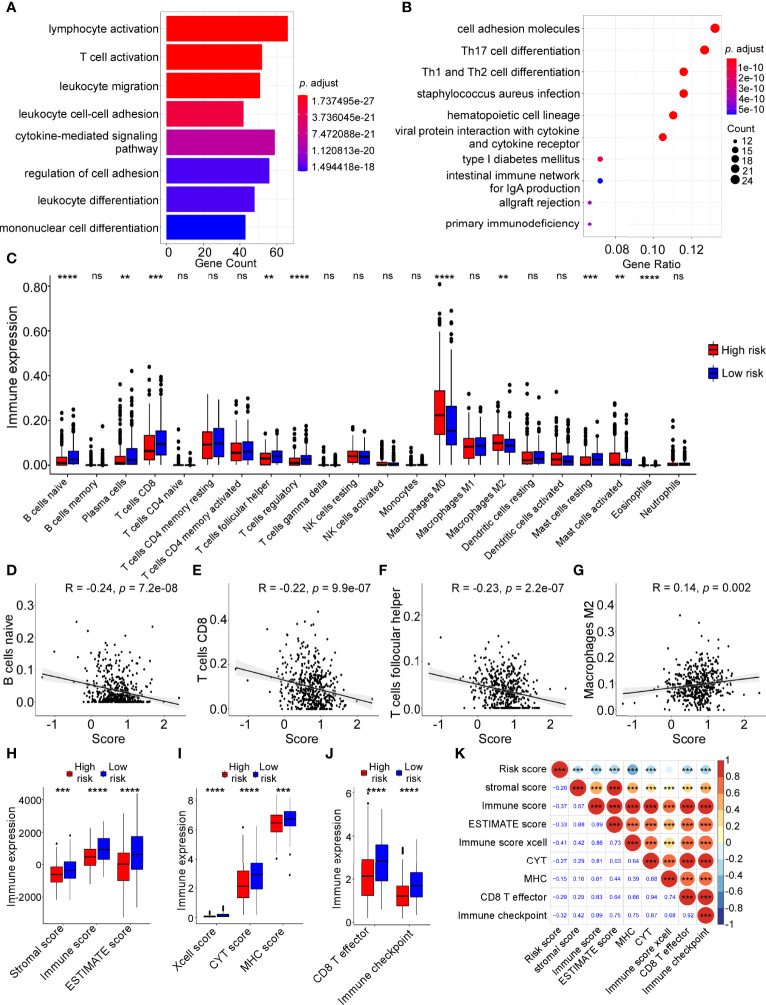
Tumor immune features and immune cell infiltration analyses in high-risk and low-risk groups. Gene ontology (GO) **(A)** and Kyoto Encyclopaedia of Genes and Genomes (KEGG) pathway **(B)** analyses were performed on dysregulated genes in different risk groups. **(C)** The infiltration levels of 22 immune cells in high-risk and low-risk groups calculated by The Cell-type Identification by Estimating Relative Subsets of RNA Transcripts (CIBERSORT) analysis (Wilcoxon test, ns: not significant; **P < 0.01; ***P < 0.001; ***P < 0.0001). **(D–G)** Linear correlation between risk score and naive B cell infiltration levels **(D)**, CD8 T cell infiltration levels **(E)**, follicular helper T cell infiltration levels **(F)**, M2 macrophage cell infiltration **(G)**. **(H)** Boxplot of ESTIMATE score in high and low risk groups (Wilcoxon test, ***P < 0.001; ***P < 0.0001). **(I)** Boxplot of xCell-immune score, CYT score, MHC score in high and low risk groups (Wilcoxon test, ***P < 0.001; ***P < 0.0001). **(J)** Boxplot of the score of CD8 T effector and immune checkpoint in high and low risk groups (Wilcoxon test, ***P < 0.0001). **(K)** Correlation of risk score with other immune features by Pearson correlation analysis.

GO and KEGG pathway analyses were carried out to explore biological functions and pathways using the dysregulated genes in the two groups. Interestingly, GO analysis revealed that immune-related functions were enriched, including lymphocyte activation, T cell activation, leukocyte migration, leukocyte cell-cell adhesion, cytokine-mediated signaling pathway and leukocyte differentiation (**Figure 6A**). Likewise, KEGG analysis showed that the differentially expressed genes were enriched in immune-related pathways, such as Th1, Th2 and Th17 cell differentiation; cell adhesion molecules; viral protein interaction with cytokine and cytokine receptor; allgraft rejection; and the intestinal immune network for IgA production (**Figure 6B**). Moreover, GSVA analysis showed low-risk group was obviously enriched in immune-related pathways, including inflammatory response, interferon gamma response, IL2/STAT5 signaling and IL6/JAK/STAT3 signaling (**Figure S3**).

To further elucidate the relationship between the risk score and immune cell infiltration in the tumor microenvironment, bioinformatic analysis was applied using the CIBERSORT algorithm. The results indicated that immune cell infiltration levels were significantly different between the high-risk and low-risk groups (**Figure 6C**). The following analysis revealed that the infiltration levels of naive B cells, CD8 T cells and follicular helper T cells were negatively correlated with the risk score, while the infiltration levels of M2macrophages were positively correlated with the risk score (**Figures 6D–G**, all P < 0.05).

Additionally, we performed other immune cell infiltration analyses and the results showed that the immune scores (stromal score, immune score, ESTIMATE score, xcell score, CYT score, MHC score, CD8 T effector score and immune checkpoint score) in low-risk group were higher compared with those in high-risk groups (**Figures 6H–J**). ADME-related risk score was negatively correlated with these immune infiltration scores (**Figure 6K**). The higher infiltration levels of immune cells, especially B cells and T cells, in the low-risk group contributed to a better immune response against cancer cells, which may explain why the low-risk group had better prognosis than the high-risk group in HNSCC.

### Immunotherapy and Chemotherapy Response Analyses

**Figure 7:**
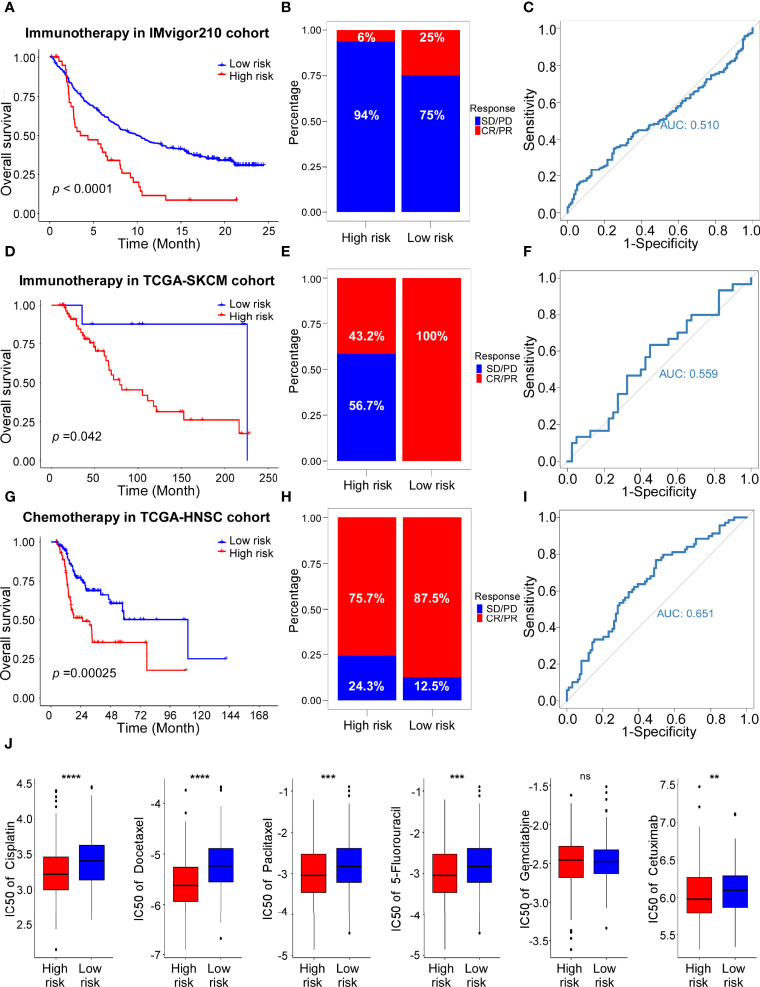
The role of risk scores in the prediction of therapeutic outcomes. **(A)** Kaplan–Meier curves for the OS of high-risk and low-risk groups in the IMvigor210 cohort. Log-rank test, P <0.0001. **(B)** Rate of CR (Complete Response)/PR (Partial Response) and PD (Progressive Disease)/SD (Stable Disease) in high and low risk groups in the IMvigor210 cohort. **(C)** ROC curve of risk score in IMvigor210 cohort on OS. **(D)** Kaplan–Meier curves for the OS of high-risk and low-risk groups in the TCGA-SKCM cohort with Immunotherapy. Log-rank test, P =0.042. **(E)** Rate of CR/PR and PD/SD in high and low risk groups in the TCGA-SKCM cohort with Immunotherapy. **(F)** ROC curve of risk score in TCGA-SKCM cohort with Immunotherapy on OS. **(G)** Kaplan–Meier curves for the OS of high-risk and low-risk groups in the TCGA-HNSC cohort with Chemotherapy. Log-rank test, P =0.00025. **(H)** Rate of CR/PR and PD/SD in high and low risk groups in the TCGA-HNSC cohort with Chemotherapy. **(I)** ROC curve of risk score in TCGA-HNSC cohort with Chemotherapy on OS. **(J)** The estimated IC50s of clinical chemotherapeutic and targeted drugs of HNSCC in high-risk and low-risk groups. From left to right, cisplatin, docetaxel, paclitaxel, 5-fluorouracil, gemcitabine, cetuximab.

Our previous results revealed the significance of ADME-related risk score in evaluating tumor immune microenvironment. To further explore the relationship between the ADME score and benefit of immunotherapy, we collected two external cohorts (IMvigor210 and TCGA-SKCM) which received immunotherapy and performed following analyses. The results showed that patients with low-risk scores had better prognosis (**Figures 7A, D**) and response to immunotherapy (**Figures 7B, E**), indicating that the low-risk group was more likely to benefit from immunotherapy. ROC curves also proved the efficacy of the ADME-related risk score in predicting responsiveness to immunotherapy (**Figures 7C, F**).

In addition, the predictive value of the ADME-related signature for chemotherapy was evaluated using the TCGA-HNSC cohort. Consistent with the results in the cohorts received immunotherapy, patients with low-risk scores showed longer survival and better response (**Figures 7G, H**). ROC analysis confirmed the predictive efficacy of the ADME signature for chemotherapy response (**Figure 7I**). Subsequently, we performed the “pRRophetic” algorithm predict the sensitivity to the 6 chemotherapeutic and targeted drugs. The results indicated that IC50s of cisplatin, docetaxel, paclitaxel, 5-fluorouracil and cetuximab were significantly different between the high-risk and low-risk groups (**Figure 7J**). In conclusion, we identified a novel ADME-related gene signature to predict immunotherapy and chemotherapy response of patients in HNSCC.

## Discussion

HNSCC, as the sixth most prevalent malignant tumor, exhibits poor clinical outcomes because of local recurrence and metastasis (28). Identification of novel predictive biomarkers in HNSCC is needed for developing individualized therapy (29, 30). In the present study, a novel ADME-related prognostic model was constructed based on the TCGA training cohort and its robustness was validated by the internal TCGA validation cohort and an external GEO cohort (GSE102349). The risk scores were calculated by the LASSO algorithm to predict the patients’ prognosis in HNSCC. The analysis indicated that patients in high-risk group exhibited shorter OS and DFS, both in the training and validation cohorts. ROC analysis confirmed the predictive efficacy of the 14-gene signature for survival of the two cohorts. The above findings suggest that our ADME-related risk score was a valuable predictor for the prognosis of HNSCC patients.

More than half of ADME genes (160/298, 53.6%) are dysregulated in HNSCC according to our analysis, indicating that ADME genes may play important roles in cancer development and progression. Here, we identified 14 ADME genes to establish the prognostic model and these genes were classified into 4 groups: phase I drug-metabolizing enzymes (CYP2D6, CYP8B1, CYP7B1, ALDH2, CYP4F12), phase II drug-metabolizing enzymes (UGT1A10), transporters (ABCB1, ABCB4, ABCB8, SLCO1B1, SLCO1B3, SULT1B1) and modifiers (PPARG, SOD1).

Recent research has reported that these ADME genes participate in tumorigenesis. Depletion of CYP2D6 influences the genes involved in EMT, oncogenesis and immune-related pathways and acts as a biomarker for drug resistance in non-small cell lung cancer (31). CYP8B1 is identified as a prognostic gene for survival analysis in hepatocellular carcinoma (32). ABCB1 is regulated by N6-methyladenosine-induced ERRγ and triggers chemoresistance in cancer cells (33). ABCB8 mediates doxorubicin resistance by protecting the mitochondrial genome in melanoma (34). SLCO1B1 polymorphisms influence the estrogenic response to aromatase inhibitor treatment in breast cancer (35). PPARG activates lipid signaling pathways, and high levels of PPARG/FASN confer a poor prognosis in prostate cancer (36). SOD1 carries a housekeeping function that maintains ROS levels below a threshold, supporting oncogene-dependent proliferation in mammary tumor formation (37). Although some ADME genes were studied in HNSCC (38–41), their biological functions and molecular mechanisms remain unclear.

To explore the biological roles of ADME genes in HNSCC, functional analyses were conducted based on the differentially expressed genes between high-risk and low-risk groups. Interestingly, GO, KEGG and GSVA analyses revealed that many important immune-related functions and pathways were enriched, including lymphocyte activation; T cell activation; leukocyte cell–cell adhesion; Th1, Th2 and Th17 cell differentiation; cell adhesion molecules; inflammatory response; and interferon gamma response. The immune system plays a vital role in the evolution and progression of HNSCC by regulating the tumor microenvironment (42, 43). According to our results, ADME genes may affect metastasis, angiogenesis or growth of HNSCC through immune-related mechanisms.

Subsequently, CIBERSORT, ESTIMATE and other analyses were performed to investigate the immune cell infiltration in the tumor microenvironment between the low- and high-risk groups. The results indicated that the two groups had significantly distinct immune cell infiltration characteristics. Interestingly, we found that infiltration levels of immune cells, especially B cells and T cells, were higher in the low-risk group than that the high-risk group. The above findings suggest that the ADME-related model could estimate immune status of HNSCC patients. The higher infiltration levels of immune-activating cells contribute to a better immune response against cancer cells, which may explain the improved prognosis observed in the low-risk group.

To further evaluate the relationship between the ADME signature and benefit of immunotherapy and chemotherapy, we collected two external cohorts (IMvigor210 and TCGA-SKCM) which received immunotherapy and obtained the drug information of TCGA-HNSC. We found that patients with low-risk scores showed better prognosis and response to immunotherapy and chemotherapy compared to those with high scores. High- risk group were more sensitive to the most used chemotherapy and targeted therapy regimens (cisplatin, docetaxel, paclitaxel, 5-fluorouracil and cetuximab) in HNSCC.

There are several potential limitations in our study. First, only six patients received immunotherapy in the TCGA cohort. Thus, we explored the predictive efficacy of the ADME signature for immunotherapy response in other cancer types. Additionally, studies in different and larger populations are necessary for validating our findings in the future.

In this study, a novel ADME-related prognostic signature of HNSCC was constructed using a TCGA cohort and validated with two validation cohorts. This risk model performed well in predicting survival of patients. Furthermore, we found the relationship of ADME-related score and immune cell infiltration in the TME. The following analysis confirmed the response prediction of the ADME score for immunotherapy and chemotherapy. Our study provides a promising prognostic signature to guide individualized therapy for HNSCC patients. In addition, targeting ADME genes may reverse TME cell infiltration and transform ‘cold tumors’ into ‘hot tumors’, providing a novel insight into potential immunotherapeutic strategies and drug combination strategies for HNSCC.

## Data Availability Statement

The datasets presented in this study can be found in online repositories. The names of the repository/repositories and accession number(s) can be found in the article/**Supplementary Material**.

## Author Contributions

Conceptualization, XT, DW and XW. Data curation, XT, DW and XW. Formal analysis, XT, RLi and YW. Funding acquisition, XT and XW. Software, Supervision, RLi. Validation, FZ and RLv. Writing – original draft, XT and RLi. Writing – review & editing, XW. All authors have read and agreed to the published version of the manuscript.

## Funding

This research was funded by Guangdong Basic and Applied Basic Research Foundation (2019A1515110076, 2019A1515011427), the National Natural Science Foundation of China (82003214, 81903134, 82172673), and China Postdoctoral Science Foundation (2019TQ0368).

## Conflict of Interest

The authors declare that the research was conducted in the absence of any commercial or financial relationships that could be construed as a potential conflict of interest.

## Publisher’s Note

All claims expressed in this article are solely those of the authors and do not necessarily represent those of their affiliated organizations, or those of the publisher, the editors and the reviewers. Any product that may be evaluated in this article, or claim that may be made by its manufacturer, is not guaranteed or endorsed by the publisher.
