# Supplementary material for: Development and Validation of an ADME-Related Gene Signature for Survival, Treatment Outcome and Immune Cell Infiltration in Head and Neck Squamous Cell Carcinoma
Source: Front Immunol. 2022 Jul 8;13:905635. doi: 10.3389/fimmu.2022.905635 (PMC9304892; doi:10.3389/fimmu.2022.905635)
Supplement: Supplementary file 1 [file DataSheet_1.pdf]

Supplementary Materials

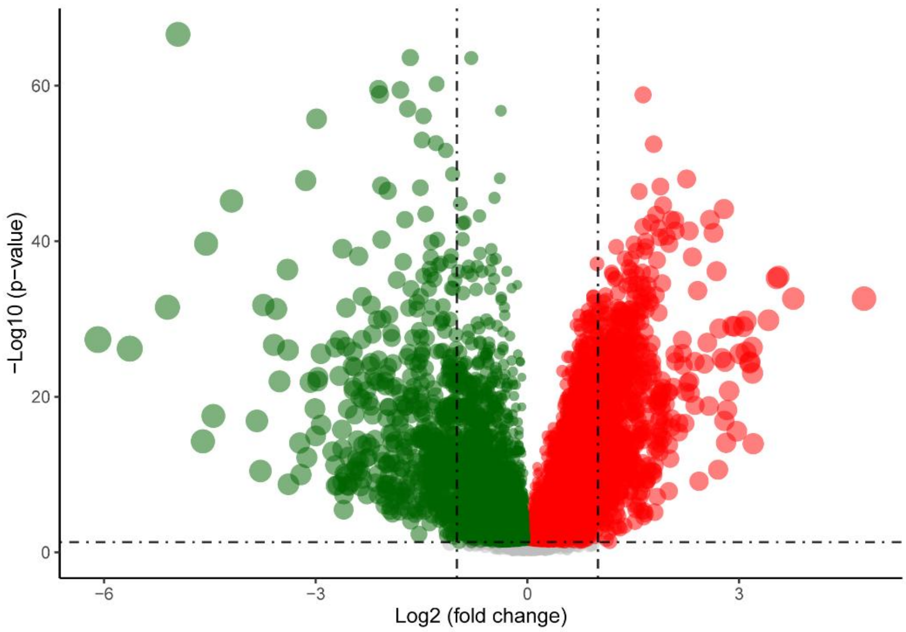

Figure S1. Volcano plot showing the gene expression profiles of HNSCC and normal tissues.

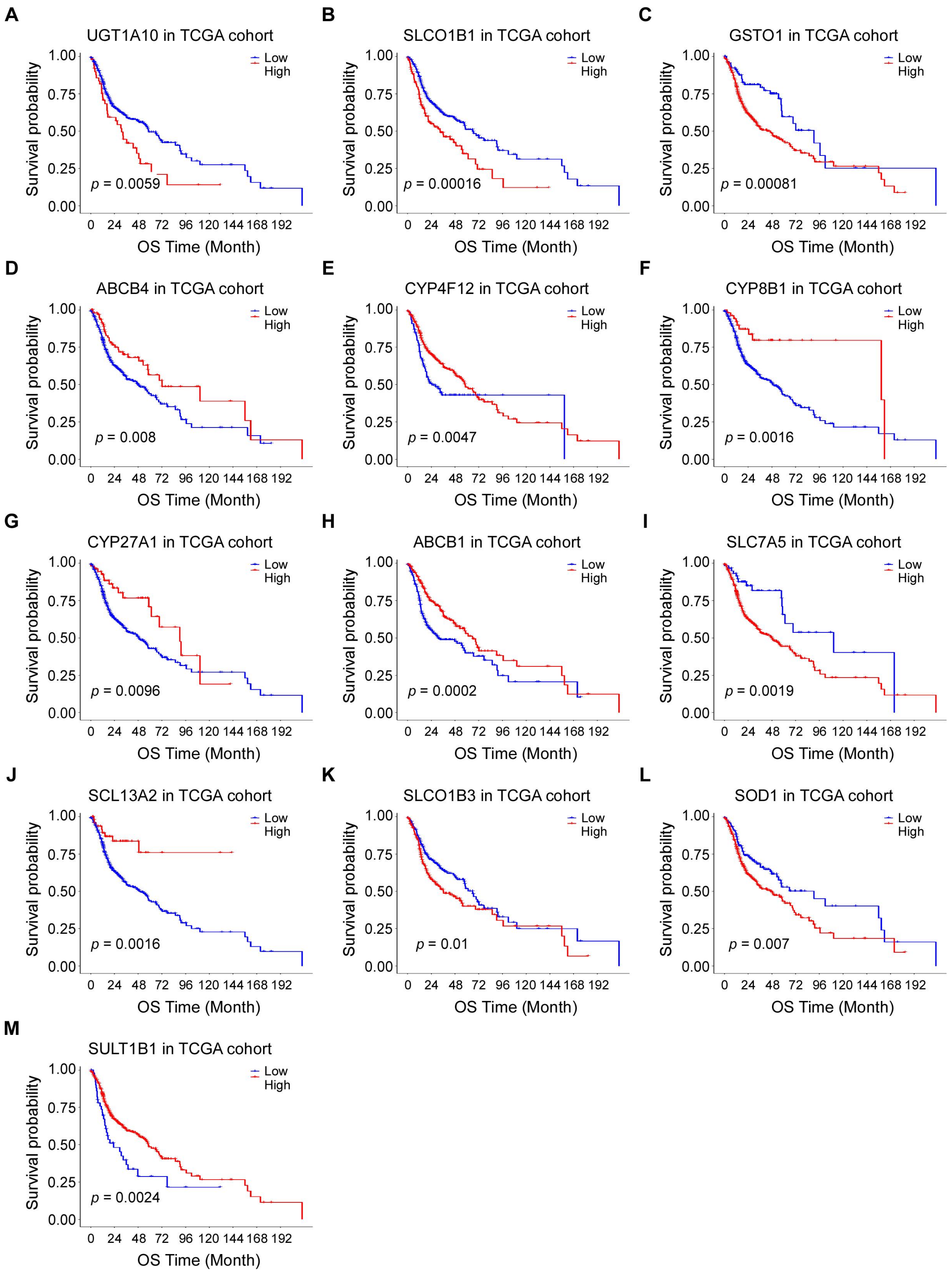

Figure S2. Kaplan-Meier curves of OS for the prognostic ADME genes in HNSCC.

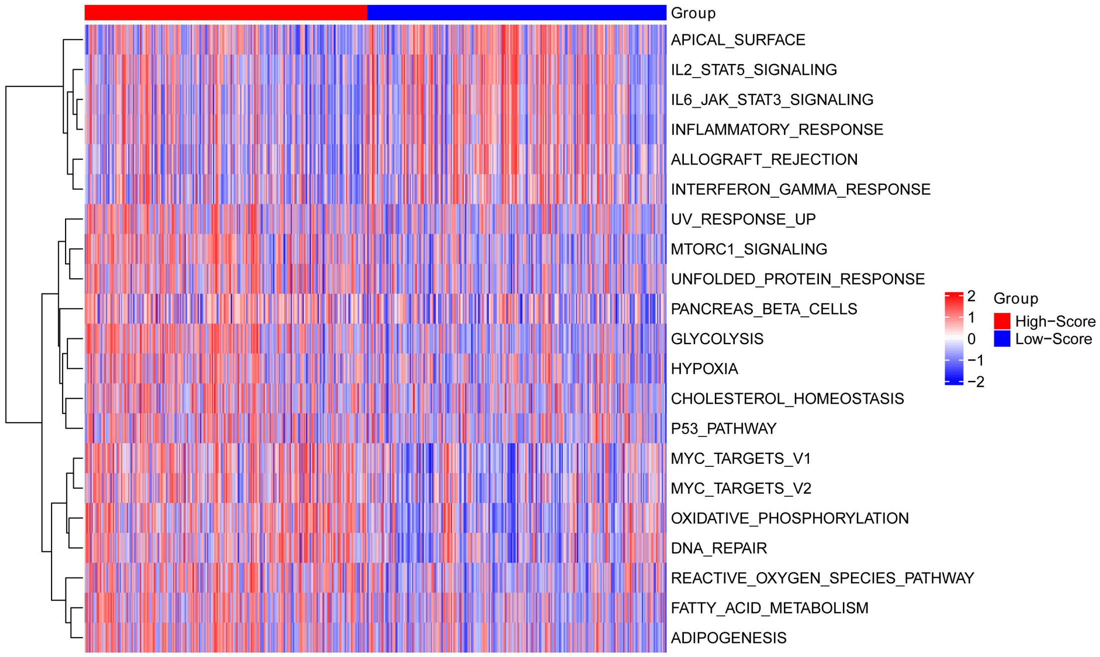

Figure S3. Gene set variation analysis (GSVA) analysis between the high-risk and low-risk groups.

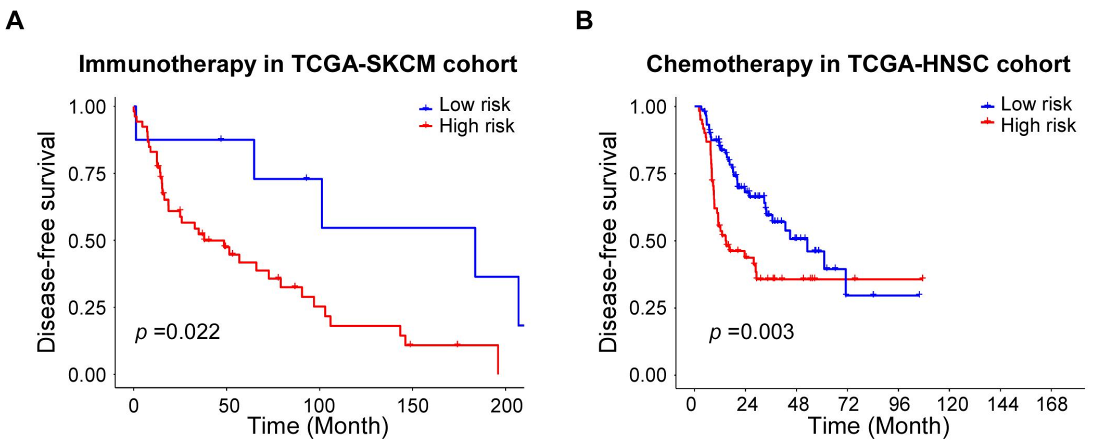

Figure S4. Kaplan-Meier curves for the Disease-free survival (DFS) of patients with high risks and low risks.
